# Fitbits for Monitoring Depressive Symptoms in Older Aged Persons: Qualitative Feasibility Study

**DOI:** 10.2196/33952

**Published:** 2022-11-29

**Authors:** Fiza Mughal, William Raffe, Peter Stubbs, Ian Kneebone, Jaime Garcia

**Affiliations:** 1 Faculty of Engineering and IT University of Technology Sydney Sydney Australia; 2 Discipline of Physiotherapy University of Technology Sydney Sydney Australia; 3 Discipline of Clinical Psychology University of Technology Sydney Sydney Australia

**Keywords:** digital mental health, Fitbit, smartwatch, smart wearable, geriatric, aging, health informatics, feasibility, usability, older aged

## Abstract

**Background:**

In 2022, an estimated 1.105 billion people used smart wearables and 31 million used Fitbit devices worldwide. Although there is growing evidence for the use of smart wearables to benefit physical health, more research is required on the feasibility of using these devices for mental health and well-being. In studies focusing on emotion recognition, emotions are often inferred and dependent on external cues, which may not be representative of true emotional states.

**Objective:**

The aim of this study was to evaluate the feasibility and acceptability of using consumer-grade activity trackers for apps in the remote mental health monitoring of older aged people.

**Methods:**

Older adults were recruited using criterion sampling. Participants were provided an activity tracker (Fitbit Alta HR) and completed weekly online questionnaires, including the Geriatric Depression Scale, for 4 weeks. Before and after the study period, semistructured qualitative interviews were conducted to provide insight into the acceptance and feasibility of performing the protocol over a 4-week period. Interview transcripts were analyzed using a hybrid inductive-deductive thematic analysis.

**Results:**

In total, 12 participants enrolled in the study, and 9 returned for interviews after the study period. Participants had positive attitudes toward being remotely monitored, with 78% (7/9) of participants experiencing no inconvenience throughout the study period. Moreover, 67% (6/9) were interested in trialing our prototype when it is implemented. Participants stated they would feel more comfortable if mental well-being was being monitored by carers remotely.

**Conclusions:**

Fitbit-like devices were an unobtrusive and convenient tool to collect physiological user data. Future research should integrate physiological user inputs to differentiate and predict depressive tendencies in users.

## Introduction

### Background

In 2021, there were approximately 280 million people diagnosed with depression worldwide [1]. Although the majority of older adults (65+ years old) are not depressed, older adults are at a higher risk of depression [2]. Eighty percent of older adults have at least one chronic health condition, which can contribute to mental illness [3,4]. Thirty percent of older adults in residential care are at risk of depression [5]. Mental illness in older adults is sometimes viewed as an inevitable reaction to changes in socioeconomic standing or increased age and has been deemed untreatable for some people [2,3]. Older adults are also more likely to be concerned about the stigma of seeking treatment for mental illness [6]. Consequently, older adults are less likely to seek help when affected by mental illness, such as depression [3].

Cost-effective smart wearables are increasingly used in the general population [7]. In 2022, there were 1.105 billion smart wearable users worldwide [7]. Of these, approximately 31 million were Fitbit users [8]. Therefore, more studies are incorporating smart wearables, particularly Fitbit devices, in health research [9-11]. A recent scoping review investigated the effectiveness and efficiency of mobile health procedures for improving physical health [12]. From 2012 to 2022, including 148 studies, there was no “one-size-fits-all approach for monitoring physical health. However, the authors found that mobile health interventions are promising for facilitating behavioral change. A similar review [13] concluded that more research was required on the effectiveness and feasibility of smart wearables for changing or assessing physical health. Using only Fitbit devices, Ringeval et al [11] assessed the effectiveness of using Fitbit devices in interventions to promote healthy lifestyle habits. Evaluating 41 studies [11], the authors concluded that Fitbit devices improved lifestyles in users. There was increased daily step counts, increased physical activity, and reduced mass in participants [11]. A further review [14] investigated the applicability of wearable devices for vital sign monitoring in outpatients and found that early detection of physiological deterioration via wearable devices likely improves patient outcomes. Although positive, on-body, potentially obtrusive sensors, such as heart rate monitors, patches, and arm bands were used, these may not be feasible for prolonged use.

The aforementioned reviews demonstrate that wearable devices can result in positive physical activity changes. Despite this, few studies have investigated how mental health might change from using smart wearables. Furthermore, very few studies have investigated older adults. This is problematic, as older adults are more likely to be apprehensive toward newer technologies [15,16]. With most studies focusing on laboratory-based settings, we proposed the use of consumer-grade smart wearables for mental health monitoring in order to bridge the gap between older people, modern technologies, and mental health [17].

### Prior Work

Smart wearables, particularly Fitbit-like devices, can be used to assist in the treatment of mental health conditions. A recent study investigated the feasibility (frequency of use) and acceptability (through follow-up interviews) of mobile health technologies to increase physical activity among users with severe mental illness [18]. Participants reported high satisfaction and increased motivation through goal setting and self-monitoring, facilitated by the mobile health technology. Another qualitative study integrated Fitbit devices with behavioral activation therapy [10]. People had more positive self-awareness, improved peer-based motivation, and improved motivation to set goals. Negative feedback included inconvenience, disinterest in participating in the study, and inaccuracy of the measurements. Furthermore, a 12-week randomized trial incorporated a non-Fitbit device (tablet) and telephone counseling to increase physical activity in older adults [19]. The intervention was feasible and accepted by the participants. It also reduced mass and increased physical activity time in older adults.

Factors such as heart rate and gait patterns can indicate depression which can be monitored using smart wearables [20,21]. Research has also validated the use of smart wearables for emotion recognition [22-25]. Smartwatches or bracelets were used and paired with external emotion-eliciting stimuli to evaluate the reliability and accuracy for emotion recognition. In laboratory-based settings, 3 studies provided participants with stimuli, including video and music clips, which were used to elicit happy, neutral, or sad emotions [23-25]. Poststimuli activities, conducted within 2 hours of emotion elicitation, included walking with a chest-mounted heart rate monitor [23]. Results showed a higher accuracy (74%) for detecting happy emotions as opposed to sad emotions. An earlier study observed walking changes 1 minute after participants received a visual stimulus to elicit an emotional response [25]. In that study, walking patterns could predict the expected emotional response 81.2% of the time. The studies show that walking patterns and smart wearables can be used to recognize user emotions. Adopting a different approach for emotion elicitation, using electrodermal activity, skin temperature, heart rate, and a Self-Assessment Manikin form, another study [22] developed an algorithm to predict emotion based on electrodermal activity signals. The algorithm accurately tagged emotions 57% of the time.

**Figure 1:**
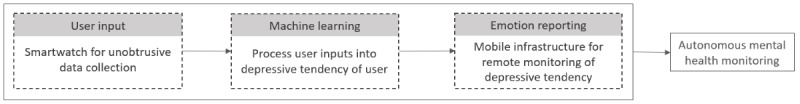
The autonomous mental health–monitoring system for older aged people (AutoMAP) framework.

In a previous study [17], we proposed an autonomous mental health–monitoring system for older aged people (AutoMAP), which provided an emotion recognition framework using minimal to no explicit user input or interaction (Figure 1). The aim of AutoMAP is to monitor the mental health of the user, without explicit user interaction (ie, not requiring the user to manually log their moods at a specific time). This framework used physiological data collected using smart wearables with the intention of predicting user mood using mood measures such as the Geriatric Depression Scale (scored from of 0 to 15, with 0 indicating no depressive tendencies and 15 indicating high depressive tendencies; scores > 9 would indicate very high depressive tendencies). The intention, when finalized, is to report depressive tendency scores to caregivers via a mobile app. In this paper, we focus on validating the feasibility (Are we able to do it?) and acceptability (Are participants willing to do it?) of our framework (Figure 1). Therefore, this paper presents the findings of a feasibility study. Although our study consists of qualitative and quantitative components, this paper describes the qualitative outcomes, centered around the feasibility and acceptability of AutoMAP. Quantitative Fitbit data, efficacy, and accuracy of the emotion recognition techniques in this framework will be presented in future papers.

## Methods

### Overview

Our study investigated the feasibility (Are the researchers able to perform the study, with the numbers required, and using the framework proposed?) and participant acceptability (How accepting are participants of the daily monitoring and questionnaires, the mobile app, and use of the mobile app?) of AutoMAP for older adults (65+ years old). Participants wore a Fitbit smartwatch for a 4-week period, completed a validated depressive symptom survey weekly, and provided a self-reported mood score daily.

### Ethical Considerations

The study was approved by the Human Research Ethics Committee (HREC) of the University of Technology Sydney (HREC reference #ETH20-4912).

### Procedures

To identify the feasibility and acceptability of our framework, we performed a hybrid inductive-deductive thematic analysis [26,27] on pre-post semistructured interviews with participants. The study protocol is accessible under the Open Science Frameworks [28].

#### Participant Recruitment and Onboarding

**Figure 2:**
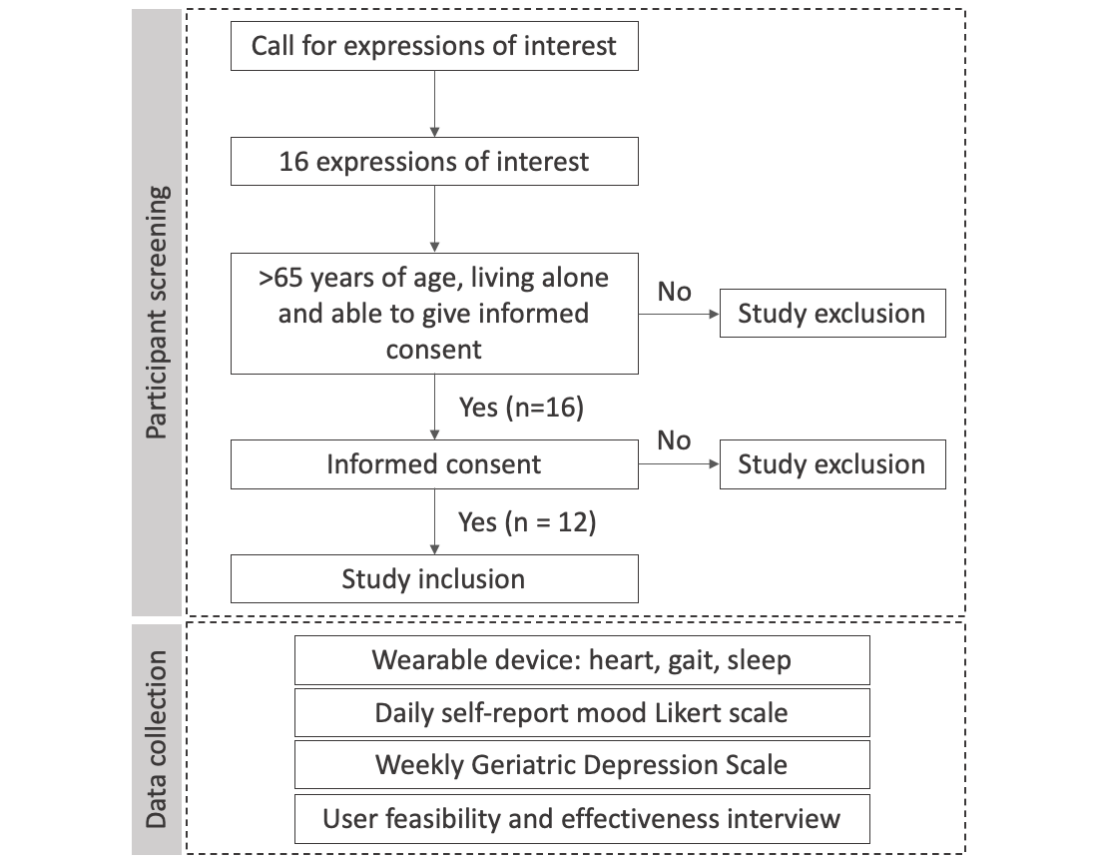
Participant recruitment flow.

Participants were recruited by word of mouth and calls for expressions of interest among senior communities and aged care facilities. There were six main inclusion criteria for the study: (1) being able to speak and understand English, (2) being aged over 65 years, (3) willing to wear a Fitbit for 4 weeks, (4) living alone with no external assistance, (5) being able to provide informed consent, and (6) having access to the internet through a computer or mobile phone. The participant recruitment flowchart is shown Figure 2. Individuals living independently were chosen for this study, as this is the target cohort for our proposed AutoMAP framework.

Similarly, there were 8 exclusion criteria for the study. These exclusion criteria included pre-existing conditions affecting sleep, gait, or heart recordings; travel plans within the duration of the 4-week period; foreseen out of the ordinary plans within the 4-week period; an inability to walk; an inability to access online surveys within the time requirement; having a history of skin rashes around the wrist area; a nickel allergy that may cause a reaction to the smartwatch charging probe; and any pre-existing diagnosis of mental health conditions. At this preliminary stage, we explicitly excluded participants with pre-existing mental health issues, as we are in the feasibility and participant acceptability stage of this research. Due to difficulties recruiting participants, 2 participants aged 64 years were also included; however they did not return for the poststudy interviews. Their data were excluded from study.

After providing an expression of interest (Figure 2), participants were provided an information sheet detailing the inclusion criteria and requirements during the 4-week period. Twelve participants were deemed eligible and were sent a participant pack. Participant packs consisted of five components: (1) a Fitbit Alta HR device, (2) a participation confirmation and information sheet, (3) a distress management resource list, (4) a simplified Fitbit user guide, and (5) a summarized task checklist. To avoid subconscious response bias, participants were blinded to the study objectives until the end of the 4-week study period. Of the 12 participants, 3 did not return for the final interviews. Figure 2 illustrates the initial screening and onboarding process at the start of the 4-week study period.

#### Wearable Device

For this study, all participants were provided a Fitbit Alta HR smartwatch. The Fitbit Alta HR smartwatch was used due to its cost-effectiveness for the eventual end user, as well as its ease of setup and usage [4,22]. Studies have shown fair performance accuracy for similar Fitbit devices (Fitbit Charge 2), with a heart rate estimation error of 14% [2]. A review of Fitbit-centered sleep studies also showed sensitivity values of 0.95-0.96 and specificity values of 0.58-0.69 for detecting sleep stages [29].

Participants were required to wear the device at all times during the 4-week period, except during showering. As the study required sleep data, participants wore the device when sleeping. The Fitbit Alta HR takes approximately 2 hours to fully charge. To ensure minimal data loss, we recommended that participants charge the Fitbit before they showered. To limit further inconvenience, we did not set fixed times for recharging.

#### Participant Protocols

Participants were provided a list of resources in case of emotional distress during the 4-week study period. Although introductory interviews were basic questions about the everyday lives of the participants, there were protocols to terminate the interview session and stop participation if a participant became distressed. Participants were informed that they were allowed to withdraw from the study at any point.

#### Researcher Interventions

Researchers could advise participants to seek assistance from a resource list or medical professionals. Participants were provided with contact information of the research team.

#### COVID-19 Protocols

The study took place in zero-contact settings to accommodate COVID-19. Participant document packs and devices were sent via post, and all interviews were held via phone.

### Data Acquisition and Analysis

#### Questionnaires

Participants completed an online daily questionnaire consisting of a self-report mood rating Likert scale, open questions on activity and food preferences (diversion questions away from depressive symptoms), and an optional section to add details of any out-of-the-ordinary events in the preceding 24-hour period. For the mood scale, participants were asked “How would you rate your mood in the past 24 hours,” rated on a scale from 1 to 10, once per day. Diversion questions, such as “What do you feel like eating?” and “What do you feel like doing?” were added to the daily mood reported questionnaire to reduce response bias.

Participants also completed an online 15-item Geriatric Depression Scale (GDS-15) once per week [30]. This is a short form of the GDS and consists of 15 yes or no questions with a single-point score for each response, which can indicate depression [31]. The participants also completed the GDS-15 questionnaire online during the introductory briefing. These responses were used as the baseline GDS scores to exclude participants (if GDS scores were >10) and for data labeling to train and test a machine learning algorithm that is part of the AutoMAP framework. As this stage is part of the quantitative phase of the research, it is not detailed further in this paper. Any participants scoring >6 on the GDS would be advised to attend a general practitioner consultation prior to participating in the study. Higher-scoring participants were not excluded immediately, as the GDS is a screening rather than a diagnostic tool for depression [31]. Both sets of questionnaires were administered through an online platform (Google Forms) dedicated to conducting and circulating online surveys.

#### Interviews

Two sets of interviews (Textbox 1) were performed at different times: (1) baseline and (2) on completion of the 4-week study period. Interviews were transcribed verbatim. The mean interview time was 11 minutes (range 4 minutes to 16 minutes) and were administered via phone by the primary researcher and coder (FM). Anonymized interviews were coded by a team of 3 additional coders (JG, PS, and WR) that had no interaction with the participants. Data were analyzed using hybrid inductive-deductive thematic analysis [26].

Prestudy interviews (Textbox 1) provided insight into the everyday lives of participants in the 4 weeks preceding the study and into the impact of COVID-19 on regular interactions (if any). We added the COVID-19 component to account for anomalies in participant data if they in fact did feel impacted by the pandemic. Poststudy interviews gained feedback on the study period itself. Poststudy interviews were held after the 4-week study period (Textbox 1). Participants were provided further details on the study and its objectives. To determine feasibility and acceptability, we asked the participants about their experience during the 4-week period, whether they would be interested in the autonomous mental health–monitoring system we have proposed, and what they would or would not want to see in the mobile app. Such an app would report their emotional state, for example, using clean, straightforward notifications versus a detailed dashboard view of vital signs.

To provide insight on whether participants felt any inconvenience or discomfort during the 4-week study period, we asked participants a set of questions about the Fitbit device. Questions 1 to 3 (Textbox 1) discussed the user experience. Questions 4 to 6 and 10 were designed to obtain feedback for improvement on the study design should the study be replicated in the future. Questions 7 and 8 asked participants about the likelihood of being more relaxed if their caregivers were informed of their mental health. Finally, question 9 was designed to validate the potential impact that the framework, as a whole, may have on older adults living alone.

**Figure 3:**
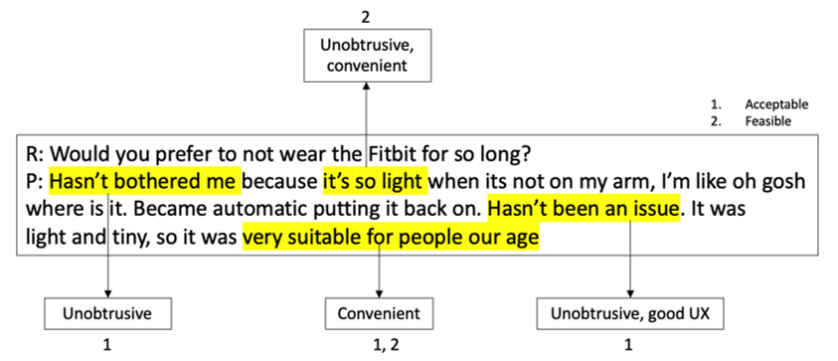
Sample excerpt analysis between the researcher and participant. P: participant; R: researcher; UX: user experience.

As the aim of this study is to evaluate the feasibility and acceptability of AutoMAP, we conducted a qualitative analysis of both sets of interviews. We assessed levels of agreement through study retention and qualitative outcomes. We performed a hybrid inductive-deductive thematic analysis [26] with 4 coders (FM, JG, PS, and WR). Our deductive thematic analysis was initially directed toward usability; however, we identified commonly mentioned subthemes to provide more insight on the interviews. In a workshop discussion, deidentified interview responses were assessed with four aims: (1) generate initial codes, (2) search for themes, (3) review themes, and (4) finally define and name the themes. Figure 3 shows an overview of how interview responses were coded and analyzed.

**Questions asked during the pre-post interviews.**

**Preintervention (introductory) interview**

1. How have you generally felt over the past four weeks?

2. How often do your caregivers visit you/you visit them (family, friends, or medical professionals)?

3. Was this the same/less/more prior to the COVID-19 pandemic?

4. Do you feel that your lifestyle or your daily life has been impacted by COVID-19 in anyway?

5. Do you feel more dependent on others now as opposed to a few years ago?

6. Geriatric Depression Scale (GDS) Questionnaire

7. Do you have any questions for us?

Follow-up questions will be asked based on their responses to the above questions.

**Postintervention (closing) interview**

1. How was your experience over the past four weeks?

2. Did anything cause you discomfort over this time?

3. How was wearing the Fitbit for 4 weeks

4. Was the daily survey inconvenient?

5. Was there any part in the survey that made you feel uncomfortable or distressed?

6. Do you have any feedback or suggestions for how we can improve the study in the future?

7. Let’s say we build a mobile app to send notifications to you or your families based on our findings, in real-time. Would that make you feel more relaxed while living independently?

8. What would you want in such an app?

9. Do you feel more at comfort when you know someone is looking after you or concerned about your inner wellbeing, regardless of whether they are with you all the time?

10. Do you have any further questions from us about our study?

11. Would your experience or answers with this study have been different prior to the COVID-19 pandemic?

Follow-up questions will be asked based on their responses to the above questions.

## Results

### General Summary

**Figure 4:**
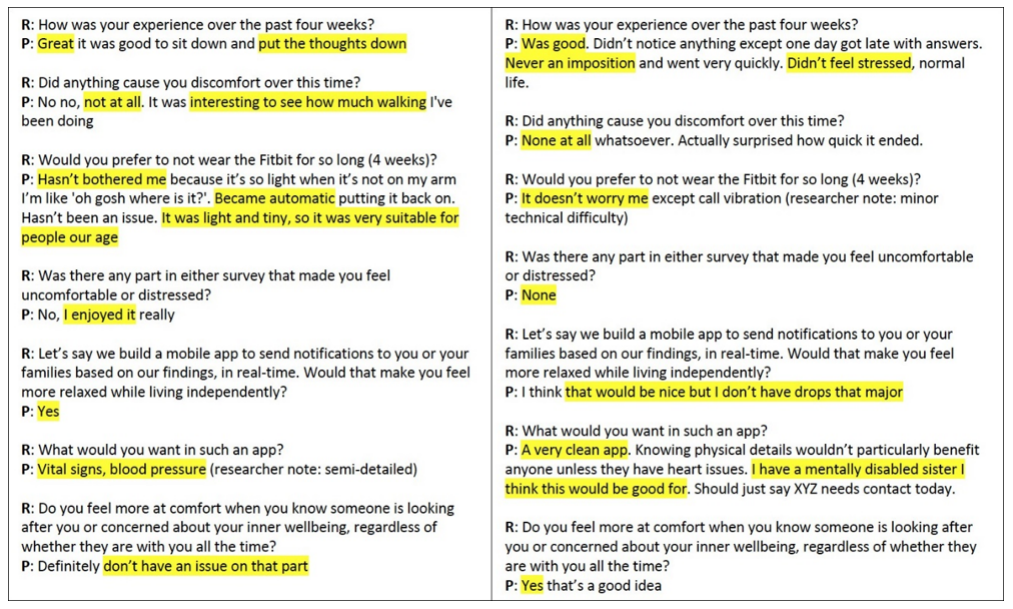
Excerpts from two researcher-participant interviews with key words highlighted by one coder. P: participant; R: researcher.

A summary of participant demographics is presented in Table 1. In all, 12 participants (mean age 68.58 years, SD 3.475 years) were interviewed prior to the 4 weeks with 9 out of 12 participants (mean age 69.11 years, SD 2.998 years) returning for a poststudy interview (75% retention). The 3 participants that did not attend the closing interviews were uncontactable, and the Fitbit data and prestudy interview responses were excluded from the study. Closing interviews were held with the remaining participants to identify the feasibility and acceptability of the framework as well as potential improvements to the proposed framework (Figure 1). Figures 3 and 4 provide an example of how interviews were analyzed. Themes were formed based on key words and the context of the overall response.

**Table 1:** Participant demographics.

ID	Sex	Age (years)	GDS^a^, mean (baseline)
1^b^	F	64	3.406 (3)
2	F	68	0.464 (0)
3	F	72	0 (0)^c^
4^b^	F	64	7.071 (6)
5	M	67	0.316 (0)
6	M	70	0 (0)^c^
7	F	68	0.974 (1)
8	F	75	6.310 (9)
9	M	71	0.4 (0)
10	F	65	0 (0)^c^
11^b^	F	73	2 (2)
12	M	66	0.448 (1)

^a^GDS: Geriatric Depression Scale.

^b^Did not return for postintervention interview and was excluded from the study.

^c^Had no change in GDS scores throughout the 4-week period.

### Preprocedure Analysis

Emergent themes from our analyses showed that participants had a positive life outlook over the preceding 4-week period. Specifically, 75% (9/12) of the participants had mostly positive emotions, with 41% (5/12) experiencing some negative emotions, 60% (3/5) of whom experienced significant negative life events. Moreover, 50% (6/12) of interviewees expressed a range of health concerns, with 83% (5/6) showing minor concerns and 17% (1/6) having major health concerns.

**Figure 5:**
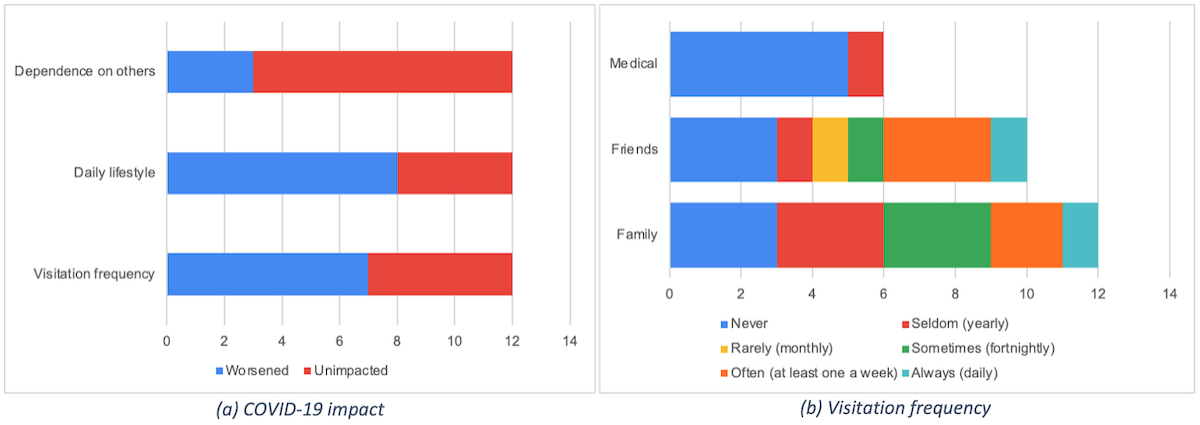
Prestudy themes: (a) COVID-19 impact on users in preceding 4-week timeframe; (b) frequency of visitation with friends, family, or medical practitioners. Bars are different lengths, as not all patients mentioned each of these items during the interviews.

All (12/12) participants had family members visiting them occasionally, while 83.3% (10/12) had more frequent visitation with friends. Only 50% (6/12) had appointments with medical practitioners, usually once per year. Visitation frequency responses (n=28) exceeded the interviewee count (n=12), as categories were not mutually exclusive; that is, participants could state visitations with any or all categories (friends, family, or medical professionals). Categorically, 57% (16/28) of the responses showed visits ranging between family, friends, or medical practitioners, once or twice per year. However, among participants that had more frequent interactions, 32% (9/28) indicated weekly interaction with friends. Of the 12 participants, 7 had less interaction since the start of the COVID-19 pandemic (Figure 5b). Additionally, 67% (8/12) of the participants felt that their lifestyle and daily lives were negatively impacted by COVID-19 (Figure 5a).

### Postprocedure Analysis

**Figure 6:**
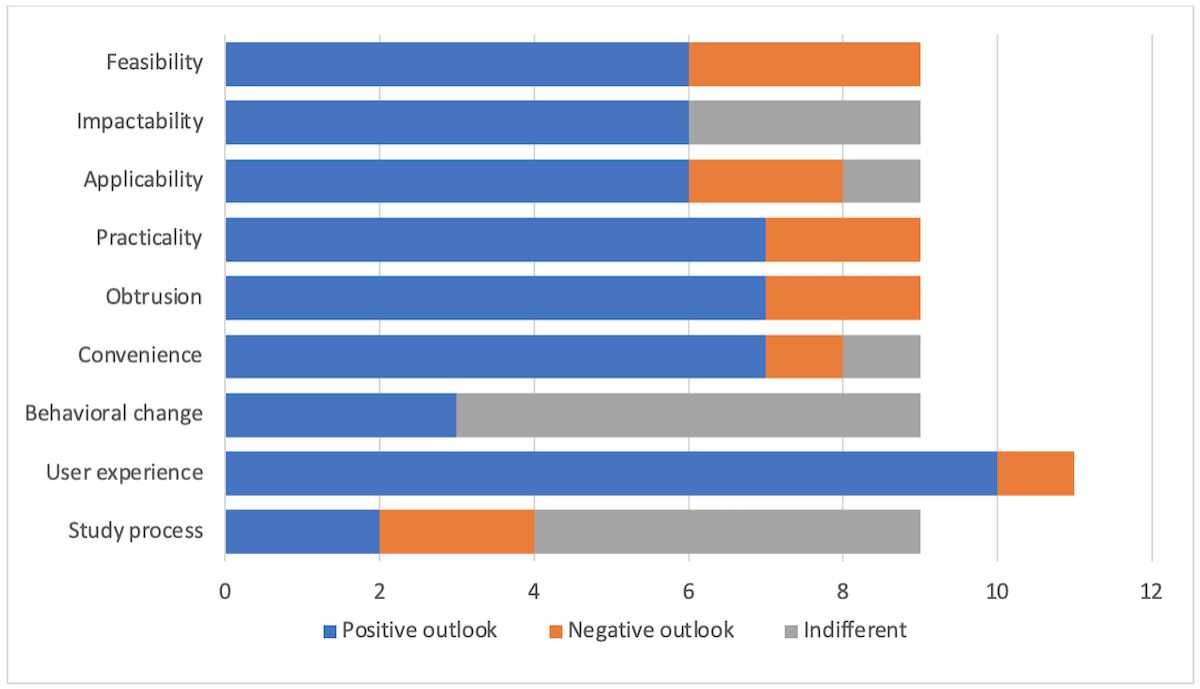
Dominant postprocedure themes for overall autonomous mental health–monitoring system for older aged people (AutoMAP) implementation and overall procedure period. User experience exceeds interview count due to repeat emphasis in responses.

Responses after the 4-week study period were generally positive: 7 out of the 9 participants were pleased with the convenience and ease of study participation as well as the limited hands-on time commitment that was required. Figure 6 illustrates a summary of the thematic analysis for the postprocedure interviews. The user experience theme exceeds 9 participants, as 2 of the 9 participants mentioned this more than once through the interview. Moreover, 33% (3/9) of participants also experienced positive behavioral changes over the 4-week study period, particularly increased awareness and motivation to exercise more.

#### Study Feasibility

Of the 9 participants, 6 (67%) felt no discomfort during the study, while 1 participant felt mild discomfort with having to wear the watch during warmer summer days. Of the 9 participants, 2 stated feeling slight frustration with having to wear the device overnight. Additionally, 7 of 9 (78%) interviewees found no inconvenience during the procedure, with the most common concerns being with the ability to report daily mood on time. Of the 9 participants, 2 felt worried about responding correctly even though there were no right or wrong answers. One participant found the diversion question “What do you feel like eating right now?” to be irrelevant, which was the purpose of the question. The procedure also resulted in behavioral change in 33% (3/9) of the participants particularly through exercise awareness and journaling during daily self-reports.

The device setup process and usage were favored by most participants, with only 1 participant having difficulty with the initial setup. Despite this, during data extraction, we found that 2 additional participants incorrectly linked their devices to the Fitbit app, which was resolved by requesting the participants to pair their devices again.

Overall, our analysis showed generally positive outcomes during the 4-week procedure. We achieved a retention rate of 75% (9/12). Some participants were seemingly aware of the study objectives although not explicitly disclosed, while others agreed it to be a good aim once they were provided more detail on our study goals.

#### Participant Usability

Further evaluating the usefulness and relevance of AutoMAP, we assessed the usability of the framework. Six of nine participants (67%) were interested in the full implementation of our working prototype and stated that they would also feel more relaxed and at comfort knowing that their mental well-being was being monitored, while the remainder felt it would be more useful for those people with major depression or diagnosed ailments. However, there were emergent themes pertaining to privacy, false positives (ie, the end user is alerted when there is no problem), and false negatives (ie, the end user is not alerted when there is a problem). These will be addressed under the mobile app requirements and constraints. Furthermore, 22% (2/9) of the participants suggested that the prototype would be more beneficial for users with specialized needs or diagnosed conditions such as Alzheimer disease, dementia, autism, Down syndrome, Asperger syndrome, or other mental health issues.

For 1 of the 9 participants, the pre-post study interviews showed a positive outlook, while the GDS scores were relatively high (mean 7). One participant that did not return for the postprocedure interviews had higher GDS scores (score ≥8) and an indifferent-to-positive outlook on life in the prestudy interview. The same participants also rated their own moods very highly (mean 8.31 and 7.8, respectively), showing that self-perceptions of emotion are not always accurate.

Of the 9 participants, 7 (67%) were pleased with the concept and potential of the prototype but raised concerns on its practicality. They suggested that the prototype may be a better fit for the special needs of persons of all ages or older adults with more serious problems, aside from general older adult populations. The envisioned end users of AutoMAP are the caregivers of older adults. However, one participant presented circumstances where the user does not have close friends or family, and the end user may not follow up on alerts.

#### Mobile App

The poststudy interviews were designed to collect suggestions and preferences for our proposed mobile app. The app would notify caregivers of the users’ depressive tendencies. Participants were provided 2 suggestions for app design: one being a neat, minimal app with only the bare essential information, such as their depressive score; and the other having more detailed information. Expanding on their preferences, 56% (5/9) of the participants preferred a clean, minimal app that would only send plain-text alerts to the app end user (caregiver) when the user showed depressive tendencies. For 2 of the 9 participants, a semidetailed app interface was preferred to a text-only interface. Another 2 participants did not specify their interface preferences but raised concerns on potential issues such as misinterpretation and alarm in case of false positives, as well as reduced privacy. If these concerns were addressed, participants would be allowed to choose the amount of information shared with their caregivers, with depressive scores being the minimum amount of information.

## Discussion

### Principal Results

Despite some participants facing minor technical difficulties during the device setup and syncing process, participants were positive about the framework. Two participants had issues with syncing the device to their Fitbit accounts through the app. Of the 2, 1 participant successfully resynced the device upon viewing an instructional video on the syncing process, while the other was unreachable despite our attempts to engage with the participant. This participant was one of the excluded participants. The participant that resynced the device was not concerned or particularly inconvenienced by the issues. The procedure resulted in positive behavioral change and improvements in physical and mental well-being. Participants commented that participation made them more aware of their physical activity, with some using the daily survey as a means of journaling, leading to mental relaxation. For people with no caregivers, we recommend a volunteer function within the AutoMAP mobile app that could allow other nominated people to check on users. This could also benefit the volunteers living through the COVID-19 pandemic through added purpose or interaction.

Participants were concerned about the possibility of false positives sent to caregivers or false negatives not being sent to caregivers. To mitigate this, we will need to train and test our algorithm to achieve high performance when determining emotion recognition from the smart watch data. When privacy is a concern, device users might choose what extra information is visible to the caregiver. This could include vital signs, depressive symptom range history, and movement patterns. We are unsure if device users should see their own emotion-level information, as it could cause subconscious bias or emotion alteration.

Although most participants favored a minimalist visual-based notification app, we will also provide options for semidetailed views for those that prefer this level of information in the app. We propose a mobile app that notifies caregivers when a user’s scores are indicative of depressive tendencies, facilitates autonomy and privacy, and allows for interface selection. Users’ scores will be determined through their physiological data and through machine learning techniques.

### Strengths and Limitations

The procedure was convenient and easy to implement from a user experience perspective. Although some participants felt mildly concerned about the device setup and compliance with weekly and daily surveys, all participants that attended the follow-up interviews were positive about the general study design and ease of participation. We blinded participants to our study aims during our preprocedure interviews and informed participants of the study aims after the 4-week procedure. This type of blinding prevented subconscious response bias. The overall impression of the study was positive, with participants reporting that the 4-week study was well conducted, easy to follow, and had no significant inconvenience to their everyday lives. Generally, the daily survey was perceived to be clear and concise although in future iterations, efforts should be made to increase the range of the mood-rating components in the survey. This could potentially allow for finer, more-detailed self-reported emotion mapping.

Our study exclusively recruited older adults with no diagnosed mental health issues. A more diverse sample could improve the future application of the AutoMAP, as well as its performance and accuracy. Although participants generally found the study favorable, some preferred to not wear the device overnight. There may also be future issues regarding the sharing of private medical user data. The latter will be assessed and detailed in future research. Despite our efforts to maintain engagement with all participants, we were unable to interview 3 of the 12 participants for postprocedure interviews. Including the perspectives of these dropouts in our analysis might have provided a different perspective on the feasibility and acceptability of the procedure and framework.

### Comparison With Prior Work

Our findings align with previous research on smart wearables for emotion recognition [23,24] or mental well-being [10,19]. These findings have shown that wearable sensors can be used for emotion recognition. Some of these studies [23,24] were in controlled settings, with deliberate emotion stimulation, where participants were not emotionally invested in the stimulus. These studies also observed participants for short time periods, meaning it is uncertain whether the stimuli used for emotion elicitation had the intended effect. By contrast, our procedure was performed in real-world settings, with participants wearing the Fitbit throughout their daily lives. Therefore, collected sensor data reflect emotions in real day-to-day life.

Promoting behavioral change through telephone counseling and smart wearables has shown promising outcomes [19]. Investigators also highlighted that competing wearable products may have different applications and accuracy, which should be accounted for in future studies using smart wearables. This study used a device from the Jawbone company, which is no longer manufacturing devices. This could render the implementation of procedures using Jawbone devices potentially invalid for long-term implementation. On the other hand, our study found feasibility for Fitbit devices in detecting preemptive depressive tendency rather than in being implemented as devices for behavioral therapy in people with diagnosed depression.

### Conclusions

We will make four modifications for the future replication of our study: (1) predefined weekly and daily survey times for all participants, (2) fewer diversion questions, (3) assisted device setup and walk-throughs, and (4) more check-ins with participants during the study period.

This study is a vital step toward validating our framework and identifying requirements for the development of our proposed mobile app. Moving forward, we will perform a quantitative analysis on participant Fitbit data, develop the mobile app, and train and test our machine learning algorithm to detect depressive tendencies using physiological inputs. The machine learning component will be integrated with the mobile app to notify caregivers if the user’s score is indicative of potential depression.

## Abbreviations

AutoMAP: autonomous mental health monitoring for older aged people
GDS: Geriatric Depression Scale
HREC: Human Research Ethics Committee
